# A Smart Voltage and Current Monitoring System for Three Phase Inverters Using an Android Smartphone Application

**DOI:** 10.3390/s17040872

**Published:** 2017-04-15

**Authors:** Mohannad Jabbar Mnati, Alex Van den Bossche, Raad Farhood Chisab

**Affiliations:** 1 Department of Electrical Energy, Metals, Mechanical Constructions and Systems, Ghent University, Technologiepark Zwijnaarde 913, B-9052 Zwijnaarde, Gent, Belgium; alex.vandenbossche@ugent.be; 2 Department of Electronic Technology, Institute of Technology Baghdad, Middle Technical University, Al-Za’franiya, 10074 Baghdad, Iraq; 3 Flanders Make, the Strategic Research Centre for the Manufacturing Industry, B-8500 Kortrijk, Belgium; 4 Department of Electrical Technology, Technical Institute Kut, Middle Technical University, Al-Za’franiya, 10074 Baghdad, Iraq; raadfarhood@yahoo.com

**Keywords:** SVCMS, voltage sensor, current sensor, Android, Bluetooth, Arduino, smart

## Abstract

In this paper, a new smart voltage and current monitoring system (SVCMS) technique is proposed. It monitors a three phase electrical system using an Arduino platform as a microcontroller to read the voltage and current from sensors and then wirelessly send the measured data to monitor the results using a new Android application. The integrated SVCMS design uses an Arduino Nano V3.0 as the microcontroller to measure the results from three voltage and three current sensors and then send this data, after calculation, to the Android smartphone device of an end user using Bluetooth HC-05. The Arduino Nano V3.0 controller and Bluetooth HC-05 are a cheap microcontroller and wireless device, respectively. The new Android smartphone application that monitors the voltage and current measurements uses the open source MIT App Inventor 2 software. It allows for monitoring some elementary fundamental voltage power quality properties. An effort has been made to investigate what is possible using available off-the-shelf components and open source software.

## 1. Introduction

Because of the increasing advances in technology, smart systems are increasingly being used. These systems allow technicians, administrators, and managers to monitor and control the performance of devices from a safe distance. The monitoring system is very important when working in the field of three phase systems; some users and companies use smart monitoring software programs [1,2,3,4]. These programs are installed on the user’s smartphone or company computers to allow employers to make decisions if there is an error.

**Figure 1:**
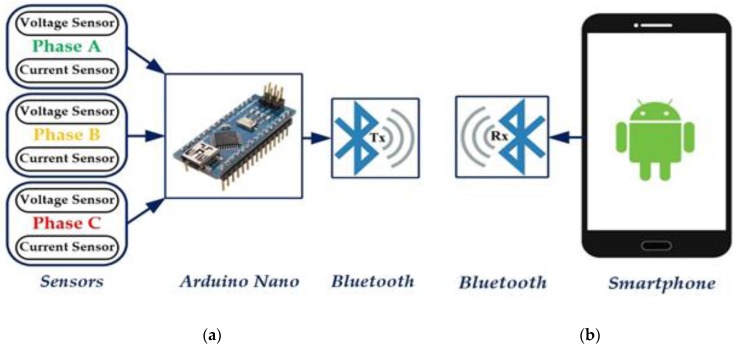
SVCMS Model: (**a**) Control system; (**b**) Monitoring system.

The main objective of this paper is to create a smart monitoring system based on an intelligent control system [5,6,7,8,9]. The proposed system is called a smart voltage and current monitoring system or SVCMS. The SVCMS is designed to monitor the performance of a three phase grid by measuring voltage and current. The SVCMS design consists of two parts; the first is the control system shown in Figure 1a. This system has been designed using the Arduino Nano V3.0 as a microcontroller to read and calculate the RMS voltage and current from sensor units [10,11]. The Arduino Nano V3.0 is an open source platform that is very cheap, flexible, and has special-purpose data processing capabilities [12]. Similar applications have been proposed for previous versions of this microcontroller [9,13,14]. The voltage sensor unit design is based on the ZMPT101B current transformer (Interplus Industry Co. Ltd., Shenzhen, China) and it amplifies the signals using a LM358 IC (Texas Instruments, Dallas, TX, USA) [15,16]. The current sensor unit is designed based on an ACS712 chip (Allegro Microsystems, Worcester, MA, USA) [17]. Both voltage and current units are isolated, very cheap, and easy to use. The last part in the control system is the Bluetooth HC-05 (Guangzhou HC Information Technology Co. Ltd., Guangzhou, China) [18,19]. This Bluetooth HC-05, is one of several types of wireless communication [20] (ZigBee, Wi-Fi, etc.) unit placed between the control system and the end user (monitoring system).

The second part of the SVCMS, seen in Figure 1b, is the monitoring system or monitoring application that is installed on a tablet or smartphone device. This application monitors the data (three phase voltage and current) received from the microcontroller. This paper uses a new application designed using MIT App Inventor 2, an open source platform from Google, that can be used to design different types of applications that can be implemented of Android smartphones or tablets [21,22,23,24].

The aim of this work was to design and implement a low cost and safe three phase measuring system and to design a smartphone application to monitor the data received from the three phase measuring system. The SVCMS has been designed to measure three phase voltages and currents for all three phase systems that have a line to ground voltage of less than 250 VAC with a current value of less than 30 A. The rest of the paper is organised as follows: Section 2 presents the relevant related research, Section 3 presents the SVCMS design of both hardware and software in detail, Section 4 discusses the practical hardware and software system results, and finally, Section 5 presents conclusions and suggests further work.

## 2. Related Work

This section discusses the system which has been designed and compares it with some related work in the same area, such as similar studies using different techniques, like different types of voltage and current sensors, wireless communication technology, type of microcontroller, and monitoring systems.

The SVCMS that has been designed in this research consists of voltage and current sensors for a three phase system, an Arduino Nano V3.0 microcontroller (electronics_lee Co. Ltd, Wuxi, China), Bluetooth HC-05 as the wireless communication system, and a new Android smartphone application designed to monitor the measured values. Table 1 shows a list of the devices used in other proposals in this area.

## 3. SVCMS Design

The SVCMS design in Figure 1 consists of two parts: the control system (practical system) and software system (the program of the microcontroller and smartphone application).

### 3.1. Control System (Hardware Design)

The control system of the SVCMS in Figure 1a has been designed to measure the voltage and current of a three phase system. Then the microcontroller calculates the RMS values to be sent to the smartphone application using Bluetooth as the wireless communication method. The practical module of the control system (SVCMS) includes the following parts:

1. Voltage sensor unit
2. Current sensor unit
3. Microcontroller unit
4. Wireless communication unit

**Figure 2:**
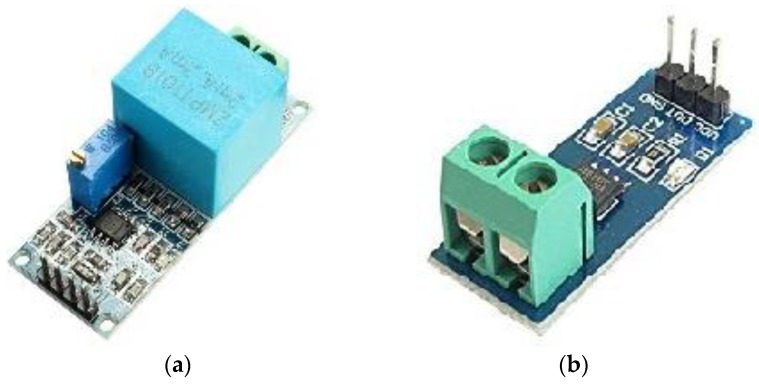

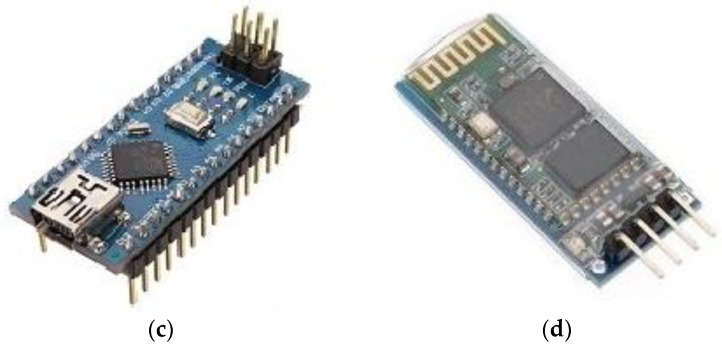
Hardware components: (**a**) voltage sensor unit; (**b**) current sensor unit; (**c**) Arduino Nano, (**d**) Bluetooth HC-05.

The four units of the hardware control system are shown in Figure 2.

#### 3.1.1. Voltage Sensor Unit

**Figure 3:**
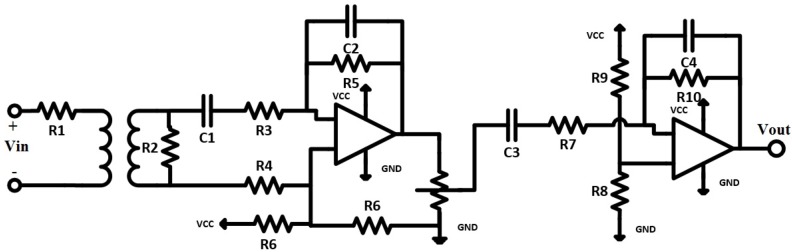
Voltage sensor circuit—A band pass (~50 Hz).

The voltage sensor circuit that is shown in Figure 3 is designed to measure the maximum AC voltage that is less than 250 VAC based on components in Figure 2a. This circuit uses a differential attenuator after a 230 VACrms with tolerance less than 5 VACpp. The output waveform (5 VAC) of the circuit is riding on DC voltage as an offset (about 2.5 V) and the amplitude can be adjusted by potentiometer but not greater than 5 V. The output of the circuit is connected directly to the ADC pin of the Arduino microcontroller.

**Figure 4:**
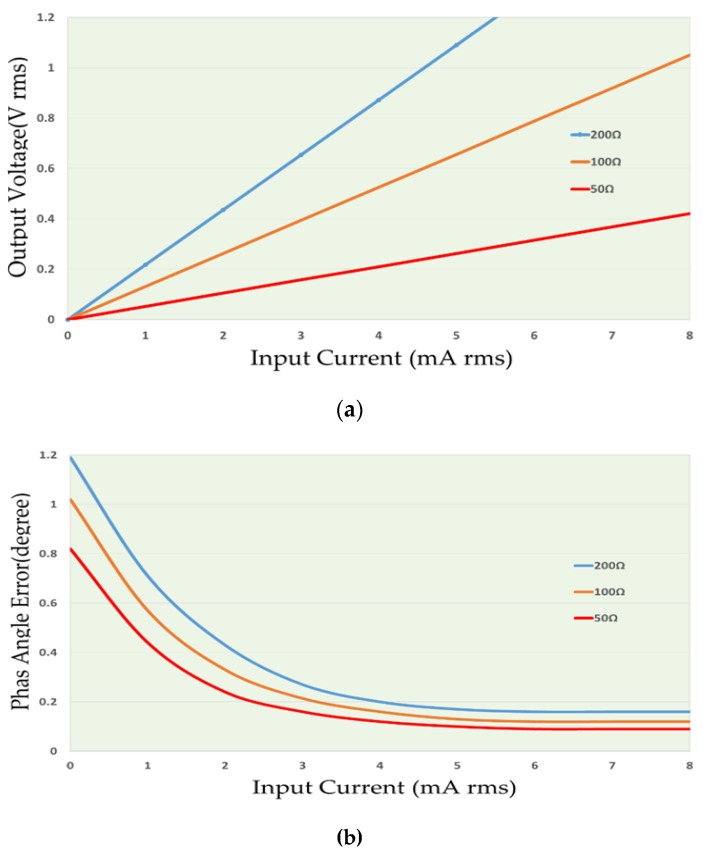
Output characteristics of ZMPT101B [15]: (**a**) output voltage and input current; (**b**) phase angle and input current.

The voltage sensor circuit design in Figure 3 is based on three stages:

1. A ZMPT101B current transformer (Interplus Industry Co. Ltd., Shenzhen, China) [15] with low impedance load (R2). The ZMPT101B is a small size current transformer with good consistency and isolation for voltage measurements. The main properties of the transformer are explained in Table 2 [15] and the output characteristics are shown in Figure 4. Two curves show in the Figure 4 depended to the input resistance of ZMPT101B, Figure 4a shows the relation between the RMS input current and RMS output voltage and Figure 4b shows the relation between the RMS input current and phase angle error of the output signal (the input resistance R1 is connected in series with the transformer).
2. Two stages of bandpass-amplifier are based on LM358 IC [16]. This chip consists of two operation amplifiers with the properties of:
  - Low power consumption
  - A wide single power supply (3 V to 32 V)

#### 3.1.2. Current Sensor Unit

**Figure 5:**
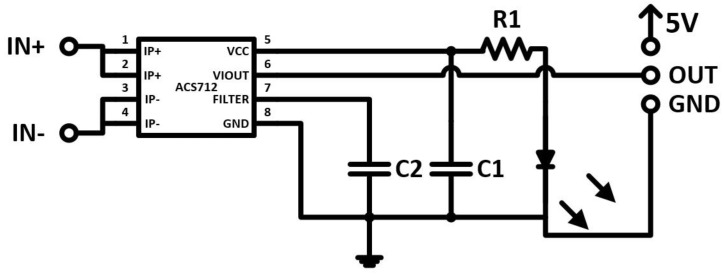
Current sensor circuit.

The current measuring circuit, shown in Figure 5, is based on the Allegro ACS712 IC sensor [17]. The ACS712 IC is a linear current sensor used for measuring AC and DC currents. This device comes in three types from the manufacturer according to the maximum current sensed (±5, ±20, and ±30 A). In this paper, the ACS712-30A was used as the current sensor. The ACS712-30A can measure currents up to ±30 A and with 66 mV/A output sensitivity on a +5 V DC power supply.

**Figure 6:**
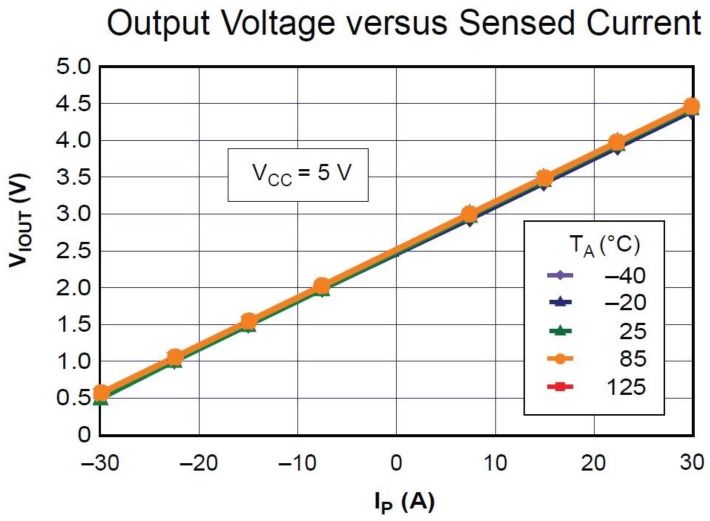
The ideal curve of the measuring current of ACS712 (I_max_ = 30 A) [17].

From data sheet [17], Figure 6 shows the curve of relation between the DC input voltage and measuring current. The main properties of the ACS712 chip are shown in Table 3.

#### 3.1.3. The Microcontroller

**Figure 7:**
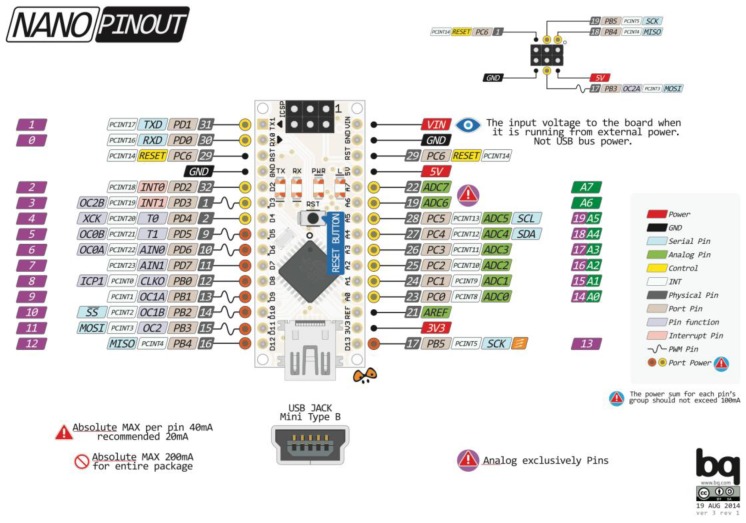
Pin diagram of Arduino Nano [28].

As previously mentioned, the main component of the control unit is the Arduino development board. The Arduino Nano V3.0 board, shown in Figure 1c, is an open source electronics prototyping platform based on V3.0 on the ATmega328, flexible hardware and software. All manufacturer properties are shown in Table 4, while the pins diagram is shown in Figure 7.

The A/D converter of Arduino Nano is 10-bit and the measuring current of the circuit is 30 A (ranging from −30 to +30) A. From the above data (Table 2 and Table 3), the quantisation noise of the A/D converter can be calculated by Equation (1):

(1) $$QN=\frac{I_{m}}{2^{N}-1}=\frac{60}{2^{10}-1}=0.058651\approx 58.651\;\mathrm{mA}$$

where *QN*: quantisation noise; *I_m_*: measuring range; *N*: A/D bits of the converter.

#### 3.1.4. Wireless Communication

The Bluetooth HC-05 module shown in Figure 1d was used as the wireless communication device between the microcontroller and the end user (smartphone device). This model is connected directly to the Arduino Nano V3.0. The Bluetooth HC-05 properties are shown in Table 5.

**Figure 8:**
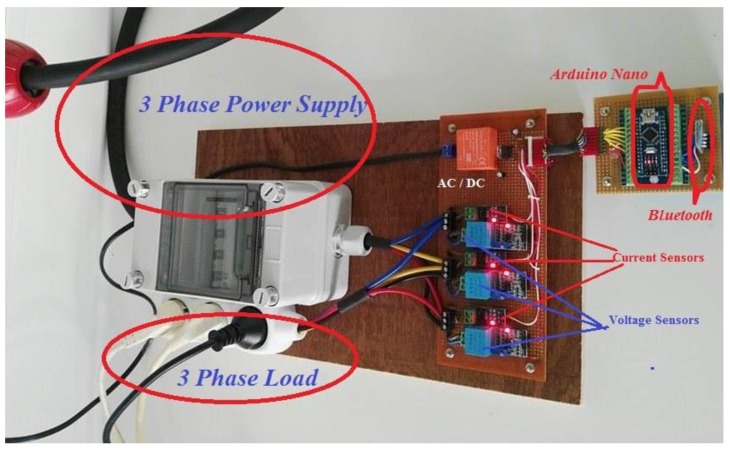
The SVCMS hardware system.

The final SVCMS hardware is shown in Figure 8, the system consists of four units, which are: (1) Arduino Nano V3.0, (2) three voltage sensors, (3) three current sensors, and (4) Bluetooth HC-5 as a wireless communication. In Figure 8, the connection of all components and how it works together are shown in order to yield the data and also shows how the data is read by the oscilloscope to check the system for errors or mistakes during the connection. The approximate total cost of the SVCMS model using parts acquired from eBay stores, as presented in Table 6, was 25€.

### 3.2. Monitoring System (Software Design)

Two types of program are used in this work, the first one is for the Arduino microcontroller to read the data from the voltage and current sensors and send the results by Bluetooth to the end user. The second one is for monitoring the received data from the microcontroller. The monitoring system or monitoring software of the SVCMS is installed on an Android smartphone or a tablet as shown in Figure 1b. This application is designed to monitor the data (three phase voltage and current) received from the microcontroller.

#### 3.2.1. Microcontroller Program

**Figure 9:**
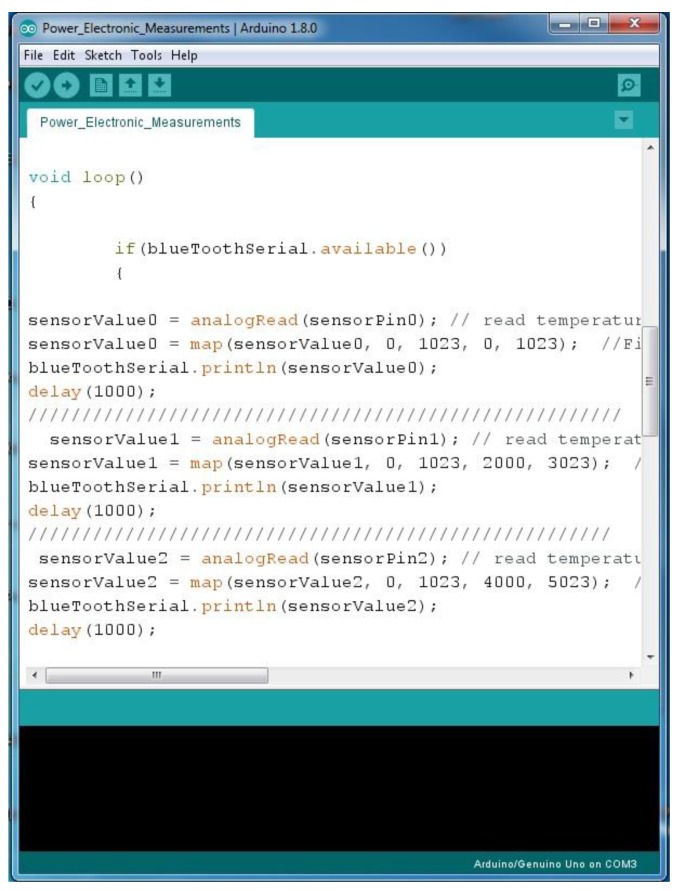
Main microcontroller program.

Figure 9 shows the main open source platform of the Arduino Nano microcontroller and shows the microcontroller codes to read the data from the voltage and current sensor units, and then send the data to the end user. This program is written in an Arduino platform and then uploads it to the Arduino board using the USB connection. In this part of the program it can be seen that the program checks the availability of the Bluetooth device to see if it is connected or not in order to read the result and send this data to the smartphone via the Bluetooth communication protocols.

#### 3.2.2. Monitoring Program

**Figure 10:**
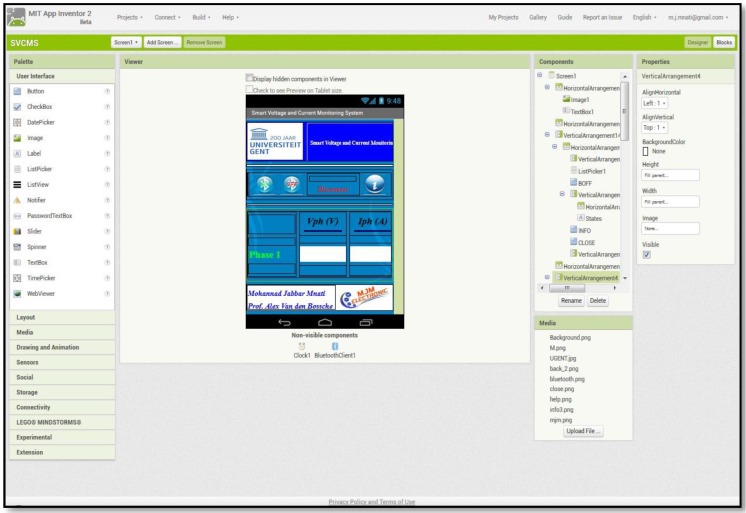
Main screen of MIT App Inventor 2.

The voltage and current for three phases are monitored by the android smartphone application. This application developed by the MIT App Inventor 2, as open source platform available from Google as online software for Android project applications. The main screen of the MIT App Inventor 2 is shown in Figure 10. This screen consists of component sets and program tools. These components include some visible items on the final smartphone application (e.g., texts, buttons, labels, etc.) and invisible items like the database, wireless tools, etc.

## 4. Discussion and Results

There are more methods for controlling and monitoring a three phase circuit depending on the controller or the type of display for the results of the voltage and current. In this paper, a new method for monitoring and displaying the three phase system is given; this method is called the smart voltage and current monitoring system, or SVCMS, where a smartphone is used instead of traditional methods like an LCD display or an analog method for monitoring and displaying the results. It consists of two major parts which are the control and monitoring parts. The control part has four branches which are: a voltage sensor unit, a current sensor unit, the Arduino Nano V3.0 unit, and wireless communication unit (Bluetooth device), while the second part contains the monitoring part that monitors the voltage and current for three phases using the Android smartphone application which is written using the MIT App Inventor 2, an open source software platform available from Google for Android project applications.

**Figure 11:**
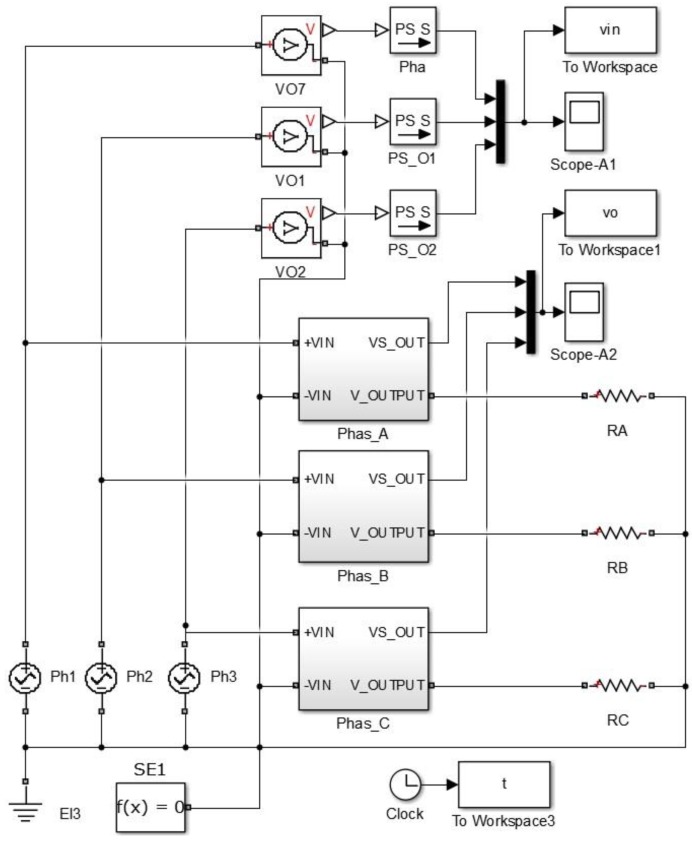
Matlab/Simulink of the three phase voltage system.

**Figure 12:**
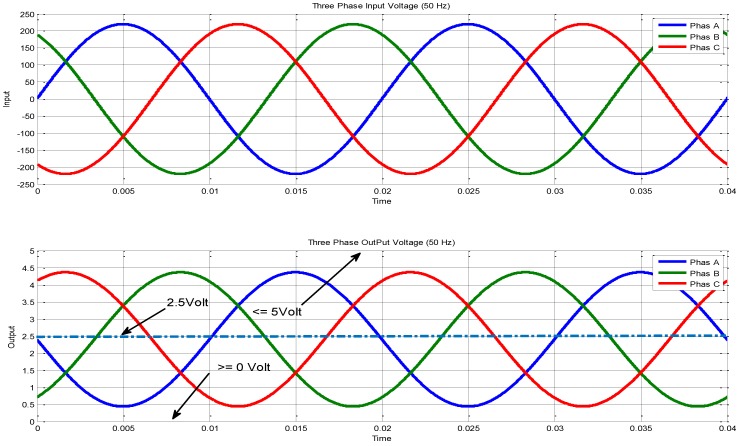
Simulation result (input and output) voltage.

Now, the control part will be discussed. It mainly consists of two sensors for measuring the voltage and current. The voltage sensor contains the transformer and two op-amps (LM358). According to Section 3.1.1 and the voltage sensor circuit in Figure 3, the Matlab Simulink simulation of the three phase voltage system is shown in Figure 11. Figure 12 shows the three phase voltage source and three voltage sensors. Figure 11 explains how the voltage can be simulated using the Matlab/Simulink in which the input voltage is shown in scope-A1 (which is about 230 V) while the output voltage can be measured in pin VS-OUT and these three phase voltages (phase-A, phase-B, and phase-C) can be shown through the scope-A2 (this voltage is about 5 V). The waveform of input and output voltage of Figure 11 is shown in Figure 12. The input voltage sine wave is offset equal to zero and upper with lower voltage of 230 V. The output voltage in Figure 12 ranges between 0 and 5 V for the microcontroller which is offset around 2.5 V. This circuit is used to reduce the voltage in order to deal with the high voltage.

The second sensor is the current sensor, as shown in Figure 5, which is based on the ACS712 IC sensor. This sensor can work under a limit of 30 A with 66 mV/A sensitivity with +5 V DC power supply. Figure 6 explains the relation between the output voltage and phase current and it can be noticed that the relation is linear under different temperature conditions from −40 °C to 125 °C; this relation increase is exactly linear and that means the increase in current taken will lead to an increase in the voltage output. Also one can notice that the change in temperature will not affect the activity of this circuit.

**Figure 13:**
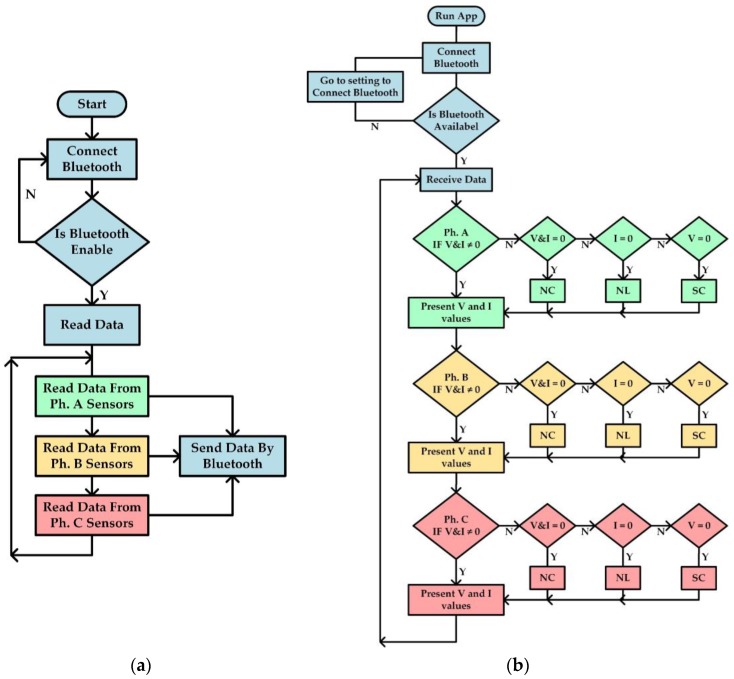
Flowchart of SVCMS: (**a**) Flowchart of Arduino Nano V3.0 Software; (**b**) Flowchart of Android application Software.

The control and software flowchart of the SVCMS is shown in Figure 13. According to Section 3.2, the flowchart in Figure 13a shows how the control system works (reading data from sensors and sending this data after calculating the RMS values by Arduino Nano through the Bluetooth HC-05 to the end user). Figure 13b shows the flowchart of the smartphone application program and how to receive data by smartphone Bluetooth from the microcontroller (control system) and then monitor the results. It can be noted that the smartphone checks the Bluetooth device to see if it is active or not in order to receive the data from the Bluetooth device HC-05 that is connected with Arduino Nano. After reading the data it will check every phase and will begin with phase A. If the voltage and current have values and is never equal to zero then it will display these values, while if the voltage is zero then it will display the (SC) and with current equal to zero display (NL) and with together equal to zero then display (NC). After that the program will check the second phase (phase B) and repeat the same steps for phase A, then go to phase C and repeat same procedure for the other two phases. Finally, it will return to “receive data” and repeat the same procedure in order to update the reading and display the new data received.

According to the Matlab/Simulink waveform in Figure 12, the Arduino Nano V3.0 calculates the RMS Value for voltage and current by Equations (2) and (3). The three phase voltage is presented in Equations (4)–(6):

(2) $$V_{ph}\;\left(RMS\right)\;=\;\sqrt{\frac{1}{T}}*\int_{0}^{T}V_{ph}{\left(t\right)}^{2}dt$$

(3) $$I_{ph}\;\left(RMS\right)=\;\sqrt{\frac{1}{T}}*\int_{0}^{T}I_{ph}{\left(t\right)}^{2}dt$$

where *T* = 1/frequency

(4) $$V_{A}=V_{m}sin\theta$$

(5) $$V_{B}=V_{m}sin\left(\theta -120\right)$$

(6) $$V_{C}=V_{m}sin(\theta +120)$$

**Figure 14:**
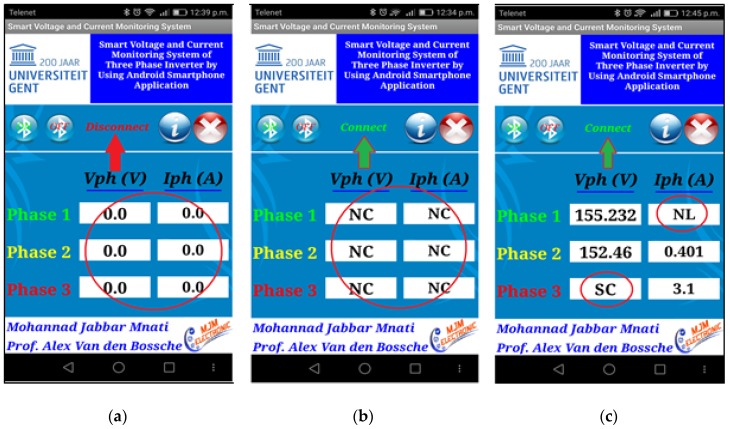
The SVCMS android application: (**a**) control system disconnected; (**b**) control system connected (no measuring signals received); and (**c**) all states of measuring sensors are shown.

The Android smartphone application is the fifth unit connected to the control system by Bluetooth. The android application of SVCMS is shown in Figure 14. Figure 14a shows the SVCMS Android application before it connects the Bluetooth to the control system (the status is “disconnect”) while Figure 14b explains the condition of the Bluetooth device after connecting (the status is “connect”) but no result appears in the main screen as the control system is not working (which means that there is no signal for all six sensors). Figure 14c presents all states of monitoring according to the flowchart application in Figure 12b.

**Figure 15:**
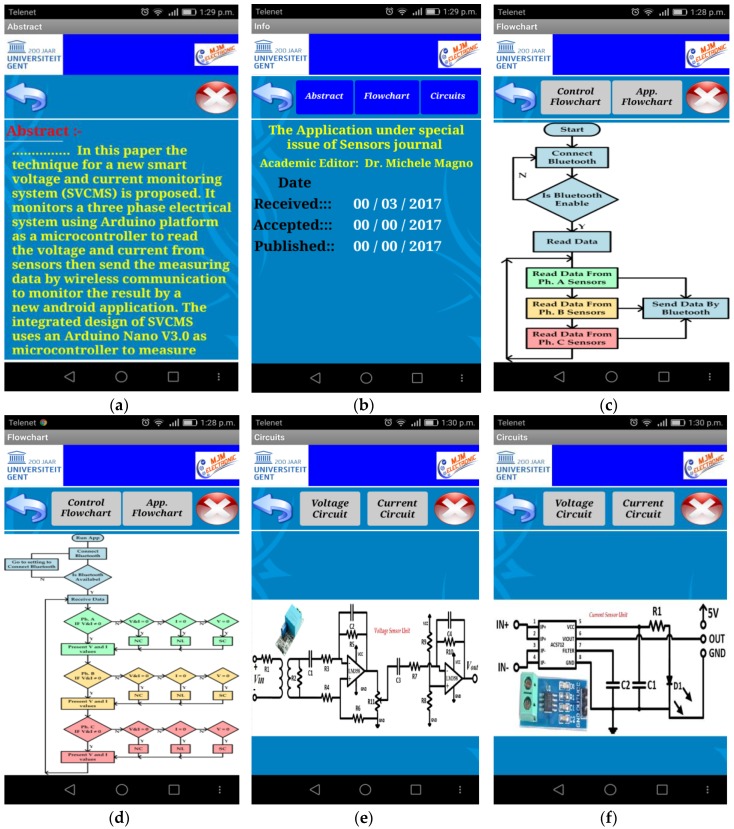
The SVCMS additional screen: (**a**) abstract; (**b**) journal information; (**c**) Arduino flowchart; (**d**) Android flowchart; (**e**) voltage sensor unit; (**f**) current sensor unit. [NOTE: The system design is based on only one SVCMS application and can be connected by the Arduino Nano at a given time].

Figure 14 explains the different cases of the connection and cases of phases. In Figure 15a the six sensor reading is zero, that means the sensor is not working because the Bluetooth device was in the off state. In Figure 15b the Bluetooth is working but the device and sensors are not connected and without receiving signals, while in Figure 15c the program is working and reading different data received from the sensors with special cases of voltage and current like “SC” and “NL”.

Figure 15 shows more screens that are programmed to explain how this system (hardware and software) works. Figure 15a shows the abstract that is written in this paper and gives a brief idea about this system for controlling and monitoring the three phases and how to it can display the results while Figure 15b shows the name of the journal where it was submitted and extra information such as the specific dates like received, accepted, and published dates. Figure 15c explains the control flow chart for the SVCMS control system and how it works while Figure 15d shows the second type of flow chart, which is the application flow chart, which works on smartphones and how it can display the results. Then Figure 15e explains the first types of sensors, which are the voltage sensors, and shows the circuit diagram of this sensor. Finally, Figure 15f shows the circuit diagram of the second sensor, the current sensor, and explains the components of this sensor.

## 5. Conclusions and Future Work

A smart voltage and current monitoring system (SVCMS), is designed and implemented to measure and monitor three phase voltages and currents. The SVCMS model is more cost effective than similar models that use heavy current transformers (CTs). It is also safer than having to measure the mains voltages very often. It is a low cost and easily applicable model for measuring and monitoring three phase system performance as compared with other models. The technician can also work with the domain like virtual reality. The monitoring system uses a new Android smartphone application designed by MIT App Inventor 2. This application receives the three phase RMS voltage and current data from the Bluetooth device (HC-05). The SVCMS has been tested successfully.

The future of smart monitoring system model and applications is to replace the Bluetooth wireless communication system by Internet of Things (IOT) technology [29,30]. This technology is used to connect the sensors and devices over the internet by allowing them to talk to us, work in applications, and interact with each other.

## Figures and Tables

**Table 1:** List of related works.

Related Work [Reference] Year	Application	Communication System	Type of Sensors	Microcontroller	Monitoring System
Gill et al. [5], 2012	Smart Power Monitoring	ZigBee	Voltage and Current	PC	PC
Aurilio et al. [1], 2014	Smart Meters	----	Voltage and Current	Arduino Shield	Ordinary
Tamkittikhun et al. [20], 2015	Power Meter Design	Ethernet Shield	Voltage and Current	Arduino Mega 2560	PC
Salamone et al. [2], 2016	Smart Lamp	Bluetooth	Temperature and Humidity	Arduino Mega 2560	Smartphone
Sung et al. [25], 2013	Smart LED	Wi-Fi + ZigBee	Light Sensor	XP-8000	Smartphone
Di Gennaro et al. [26], 2014	Monitoring System	ZigBee	pH Probe	Raspberry Pi	PC
Calderón et al. [7], 2016	Monitoring System	Cable	Temperature	Arduino Mega and PC	PC
Kim et al. [27], 2015	Monitoring System	Wi-Fi	Webcam	Embedded Linux Board and Arduino	Smartphone

**Table 2:** The main properties of ZMPT101B [15].

Parameter	Value
Turns Ratio	1000:1000
Primary and Secondary Current	2 mA and 2 mA
Dielectric Level	3000 VAC/min
Frequency Range	50~60 Hz
Phase Angle Error	≤20°, (50 Ω)

**Table 3:** The main properties of ACS712 [17].

Parameter	Value
Supply Voltage	5 V
Minimum Isolation Voltage (Input & Output)	2.1 kVrms
Sensitivity (±5, ±20 and ±30) A	(66,100, 185) mV/A
Working Temperature	From (−40 to + 85) °C
Consumed Current	10 mA

**Table 4:** Arduino Nano Properties.

Parameter	Value
Microcontroller	ATmega328
Operating Voltage (logic level)	5 V
Flash Memory, EEPROM, and SRAM	32 kB of which 2 kB used by bootloader, 1 kB, and 2 kB
Clock Speed	16 MHz
Analog I/O Pins	8
DC Current per I/O Pins	40 mA (I/O Pins)
Input Voltage	7–12 V
Digital I/O Pins	22
PWM Output	6
Power Consumption	19 mA

**Table 5:** Properties of Bluetooth HC-05.

Parameter	Value
Frequency	ISM band, 2.4 GHz
Synchronous	1 Mbps/1 Mbps
Power Supply	+3.3 VDC 50 mA
Working Temperature	(−25~+75) °C
Transmit Power	Class 2, ≤4 dBm

**Table 6:** SVCMS model cost analysis.

Component	Hardware	Quantity	Price (€)
Voltage Sensor	Single-phase AC Voltage Sensor Circuit	3	3 × 4.26
Current Sensor	Single-phase AC current Sensor Circuit	3	3 × 1.7
Wireless Communication	HC-5 Bluetooth	1	3.41
Microcontroller Board	Arduino Nano	1	3.26
Total cost from eBay stores (approximately)			24.55
